# Insignificant Response of Bacterioplankton Community to Elevated *p*CO_2_ During a Short-Term Microcosm Experiment in a Subtropical Eutrophic Coastal Ecosystem

**DOI:** 10.3389/fmicb.2021.730377

**Published:** 2021-11-12

**Authors:** Yunlan Yang, Fei Zhang, Xiaowei Chen, Huifang Li, Nianzhi Jiao, Rui Zhang

**Affiliations:** ^1^College of the Environment and Ecology, Xiamen University, Xiamen, China; ^2^State Key Laboratory of Marine Environmental Science, Fujian Key Laboratory of Marine Carbon Sequestration, Xiamen University, Xiamen, China; ^3^Laboratory of Marine Biology and Ecology, Ministry of Natural Resources, Third Institute of Oceanography, Xiamen, China; ^4^College of Ocean and Earth Sciences, Xiamen University, Xiamen, China

**Keywords:** elevated *p*CO_2_, bacterioplankton community, abundance, community composition, eutrophic coastal ecosystem

## Abstract

Ocean acidification, as one of the major consequences of global climate change, markedly affects multiple ecosystem functions in disparate marine environments from coastal habitats to the deep ocean. Evaluation of the responses of marine microbial community to the increasing partial pressure of CO_2_ (*p*CO_2_) is crucial to explore the microbe-driven biogeochemical processes in the future ocean. In this study, a microcosm incubation of eutrophic coastal water from Xiamen Bay under elevated *p*CO_2_ (about 1,000 μatm) and control (ambient air, about 380–410 μatm) conditions was conducted to investigate the effect of ocean acidification on the natural bacterioplankton community. During the 5-day incubation period, the chlorophyll *a* concentration and bacterioplankton abundance were not significantly affected by increased *p*CO_2_. Hierarchical clustering and non-metric multidimensional scaling analysis based on Bray-Curtis similarity among the bacterioplankton community derived from the 16S rRNA genes revealed an inconspicuous impact of elevated *p*CO_2_ on the bacterial community. During the incubation period, Proteobacteria, Bacteroidetes, Actinobacteria, Cyanobacteria, and Epsilonbacteraeota were predominant in all microcosms. Despite the distinct temporal variation in the composition of the bacterioplankton community during the experimental period, statistical analyses showed that no significant difference was found on bacterioplankton taxa between elevated *p*CO_2_ and control, indicating that the bacterioplankton at the population-level were also insensitive to elevated *p*CO_2_. Our results therefore suggest that the bacterioplankton communities in the fluctuating and eutrophic coastal ecosystems appear to be adaptable to the short-term elevated *p*CO_2_.

## Introduction

Human activities have triggered substantial changes in global climate systems with a preternatural rate over the past two centuries, leading to massive CO_2_ absorption by the world’s oceans and a reduction in the pH of seawater which is known as ocean acidification (Caldeira and Wickett, 2003; Orr et al., 2005). The partial pressure of atmospheric CO_2_ (*p*CO_2_) has increased by nearly 40% from the preindustrial period to the present day (about 400 μatm) and is predicted to reach approximately 1,000 μatm by the end of this century (Caldeira and Wickett, 2003; IPCC, 2013). Ocean uptake of CO_2_ changes the equilibrium of the carbonate system, and the continued release of anthropogenic CO_2_ may lead to another 0.3–0.4units decline in seawater pH globally by 2100 (Orr et al., 2005; IPCC, 2013). As the most complicated and productive ecosystems, coastal oceans generally consist of diversiform but tightly connected aquatic environments, such as rivers, estuaries, tidal wetlands, and sea margins, all of which are strongly influenced by climatic and anthropogenic factors (Cai et al., 2011; Melzner et al., 2012; Wallace et al., 2014). Recent syntheses of the air-sea CO_2_ fluxes in coastal waters suggest that CO_2_ uptake in coastal ecosystems has reached to 0.22–0.45 Pg C yr.^−1^, which is expected to affect the global carbon flux (Cai et al., 2006; Bauer et al., 2013; Regnier et al., 2013). In addition to a large CO_2_ sink for the atmosphere, ocean acidification in coastal habitats was also reported to be amplified by eutrophication and hypoxia, and these regions might be grimmer by future climate change than previously thought (Cai et al., 2011; Melzner et al., 2012; Wallace et al., 2014). Therefore, the subsequent effects of ocean acidification on coastal life have become one of the most important issues.

Microorganisms exist everywhere and are abundant in density and genetic diversity (Azam and Worden, 2004; Azam and Malfatti, 2007). It has been estimated that microorganisms account for more than two-thirds of marine biomass, despite their tiny size (Bar-On and Milo, 2019). Furthermore, they are key components of the marine food web and play crucial roles in marine ecosystem function and carbon cycling (Azam, 1998; Jiao et al., 2010). The responses of microorganisms to climate change (e.g., ocean acidification) will be pivotal for the marine food web and the biogeochemical cycle in the future ocean. The responses of cyanobacteria, as important primary producers in the ocean, to elevated *p*CO_2_ have been investigated in terms of their growth rates, photosynthesis, carbon concentration mechanisms, and cellular affinities for inorganic carbon (Fu et al., 2007; Flombaum et al., 2013; Dutkiewicz et al., 2015; Hutchins and Fu, 2017; Ma and Wang, 2021). With a higher abundance than cyanobacteria by 1–2 orders of magnitude, heterotrophic bacteria play a major role in recycling dissolved organic carbon and nutrients through the microbial loop; however, far less studies were conducted on relationships between heterotrophic bacteria and ocean acidification than phytoplankton (Azam et al., 1983; Azam, 1998; Robinson and Ramaiah, 2011). Previous studies have shown that responses of bacterioplankton communities to elevated *p*CO_2_ are diverse and complex and even conflicting. Bacterioplankton communities were found to be susceptible to changes in *p*CO_2_ or pH in some studies that were reflected in abundance, diversity, or composition (Krause et al., 2012; Zhang et al., 2013; Xia et al., 2019; Aguayo et al., 2020; Crummett, 2020), whereas other studies reported negligible effects (Roy et al., 2013; Oliver et al., 2014; Wang et al., 2015). Take coastal ecosystems for example, microbial community from coastal ecosystem with naturally low pH (average=7.8) was still sensitive to acidification (Crummett, 2020). However, recent evidence demonstrated that bacterioplankton community from variable coastal ecosystem was relatively stable under elevated *p*CO_2_ condition (Allen et al., 2020). The relationships between bacterioplankton communities and elevated *p*CO_2_ were more likely to be associated with environmental characteristics, such as nutrients and temperature (Bergen et al., 2016; Sala and Aparicio, 2016; Allen et al., 2020; Wang et al., 2021). Nevertheless, most studies on this topic have been performed in mesotrophic high-latitude regions, especially the Arctic Ocean, while less investigations were conducted in eutrophic low- and middle-latitude coastal ecosystems.

As a subtropical coastal region, Xiamen Bay is characterized by the input of nutrient-rich freshwater from Jiulong River, intrusion of saltwater from the South China Sea, and inflow of artificial wastewater, as well as being affected by intensive human activities (Chen et al., 2013; Cai et al., 2016). The Xiamen coastal ecosystem has showed nutrient-enhanced eutrophication since mid-1990s because of its hydrographical setting (Chen et al., 2013; Cai et al., 2016; Fu et al., 2016). Analyses of the long-term variations in the concentrations of nutrients in Xiamen coastal seawater suggest that dissolved inorganic nitrogen and phosphate have increased by several fold over recent decades (Cai et al., 2016). A time series sampling investigation revealed that the concentration of dissolved organic matter has even in Xiamen coastal ecosystem (Chen et al., 2021b). And estuarine input contributed a mass of dissolved organic matter, including humic-like fluorescent dissolved organic matter, S-containing, and N-containing organic molecules, to the coastal ecosystem (Chen et al., 2021b). In addition to eutrophication, interactions between anthropogenic CO_2_ emissions and local drivers in coastal ecosystems (such as eutrophication, hypoxia, and biological activities) result in complex regulation of the pH and carbon cycle in coastal waters (Cai et al., 2011; Melzner et al., 2012; Wallace et al., 2014). Analyses of the annual variations in water quality indices in Xiamen Bay from 1986 to 2007 revealed that the mean pH values ranged from 7.86 to 8.21 and continuously decreased (Cai et al., 2016). To investigate the effects of elevated *p*CO_2_ on bacterioplankton community at such a changing environment, a 5-day microcosm incubation experiment with eutrophic coastal water from Xiamen Bay under elevated *p*CO_2_ and ambient conditions was conducted. During the incubation, the response of bacterioplankton community structure to elevated CO_2_ and the population size of bacterioplankton were continuously detected.

## Materials and Methods

### Location and Experimental Setup

**Figure 1:**
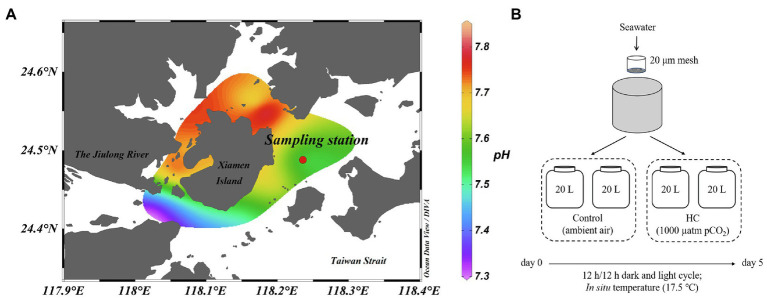
Location of sampling station **(A)** and setup of microcosm experiment **(B)**. The mesocosms were treated with two levels of *p*CO_2_, HC (about 1,000 μatm) and control (about 380–410 μatm), and incubated under dark (0 μE m^−2^ s^−1^) and light (64.4 μE m^−2^ s^−1^) cycles at approximately *in situ* temperature (about 17.5°C). Map is colored according to the pH values of seawater around Xiamen Island. Map was constructed using Ocean Data View software (version 5.3.0).

The microcosm experiment was carried out in March 2013 using surface seawater collected from a site east of Xiamen, China (24°29′47″N, 118°14′12″E; Figure 1A). Environmental parameters, such as pH, temperature, salinity, and dissolved oxygen, were measured *in situ* using a YSI Professional Plus multiparameter meter (YSI Incorporated, Yellow Springs, OH, United States). For nutrients analysis, water samples were filtered through a 0.45μm cellulose acetate filter and stored at −20°C. The nitrite (NO_2_^−^), nitrate (NO_3_^−^), phosphate (PO_4_^3−^), and silicate (SiO_3_^2−^) concentrations were analyzed according to colorimetric method with a Technicon AA3 Auto-Analyzer (Bran+Lube, GmbH, Norderstedt, Germany; Dai et al., 2008; Han et al., 2012). To determine microbial abundance, samples (1.98ml for each one) were fixed with a final concentration of 0.5% glutaraldehyde (Sangon Biotech, Shanghai, China) for 15–30min at room temperature (about 25°C), flash-frozen in liquid nitrogen, and then kept at −80°C until analysis (Marie et al., 1997).

The microcosm experimental design is shown in Figure 1B. Briefly, a total of 80L of surface seawater were collected and pre-filtered through a 20μm mesh to remove the large-size fraction. The pre-filtered seawater was then distributed into 20L polycarbonate bottles (Nalgene, United States), with approximately 60–70% of light transmittance, that served as the experimental microcosms (Ha et al., 2013). To simulate atmospheric CO_2_ concentrations currently and by the end of this century, two levels of *p*CO_2_ (ambient air was set as control, about 380–410 μatm; elevated *p*CO_2_ was set as HC treatment, about 1,000 μatm) were obtained by adjusting ambient air with CO_2_ using an enrichment device (Wuhan Ruihua Instrument and Equipment, Wuhan, China). For equilibration of the carbonate system, bubbling was continued throughout the experiment. The pH was monitored using a pH detector (Thermo Scientific, Waltham, MA, United States) during the whole incubation period (Supplementary Figure 1). The microcosms were incubated under a 12h light (64.4 μE m^−2^ s^−1^) and 12h dark (0 μE m^−2^ s^−1^) cycle at approximately *in situ* temperature (about 17.5°C), using a light incubator equipped with fluorescent lamps of 400–700nm wavelength (PGX-450B-HM, Ningbo Saifu, China). Two replications were established for the HC treatment and the control. During 5-days of incubation, subsamples were collected from all microcosms every other day to determine the chlorophyll *a* (Chl *a*) concentration and the microbial abundance and community structure.

### Measurements of Chlorophyll *a* Concentration and Microbial Abundance

For Chl *a* determination, about 100ml water samples were filtered onto GF/F filters (25mm, Whatman, Sigma-Aldrich, MO, United States) and extracted overnight at 4°C in absolute methanol. Then, each sample was centrifuged at 5,000× *g* for 10min to remove particulates, and the absorbance of the supernatant was determined using a UV-VIS spectrophotometer (Beckman Coulter, Brea, CA, United States). The Chl *a* content was calculated according to the formulae reported by Porra (2002).

Total bacterioplankton (including autotrophic and heterotrophic bacteria), virioplankton, and autotrophic picoplankton were counted with a flow cytometer (Epics Altra II, Beckman Coulter, United States; Marie et al., 1997; Jiao et al., 2002; Brussaard, 2004; Brussaard et al., 2010). After thawing, bacterioplankton samples were diluted in Tris-EDTA buffer (pH=8, Sigma-Aldrich) and then stained with SYBR Green I (10,000× final concentration in DMSO, Molecular Probes, Invitrogen, Carlsbad, CA, United States) for 15min in the dark at room temperature (about 25°C). Samples for virus counting were dyed with SYBR Green I for 10min at 80°C in the dark before analyzed. Abundance of autotrophic picoplankton (picoeukaryote, *Synechococcus*, and *Prochlorococcus*) could be directly detected by flow cytometry without dyeing. As an internal standard, 10μl of 1μm diameter fluorescent microspheres (Molecular Probes Inc.) was added to all samples before flow cytometry analysis. The data were obtained and analyzed with EXPOTM^32^ MultiCOMP software (Beckman Coulter) and FCM Express software (*De Novo* Software, version 3). Green fluorescence and side scatter were recorded and used as discriminators of bacterioplankton and virioplankton, while autotrophic picoplankton was identified in the plots of side scatter vs. red fluorescence and orange fluorescence vs. red fluorescence.

### DNA Extraction, PCR, and Sequencing

Microbial cells were collected from a 1 L sample of each microcosm for bacterial community structure analysis using membrane filtration (0.22μm-pore-size Isopore membrane, Millipore, Billerica, MA, United States). To avoid nucleic acid degradation, samples were flash-frozen in liquid nitrogen and stored at −80°C until DNA extraction. Parallel samples were mixed, and total genomic DNA was extracted using a bacterial DNA extraction kit (Tiangen DP302, Beijing, China) following the manufacturer’s instructions. Amplification, library construction, and sequencing of the extracted DNA were conducted by the Shanghai Personal Biotechnology Co., Ltd. (Shanghai, China). The V4 region of the bacterial 16S rRNA gene was amplified with the primers 520F (5'– GCACCTAAYTGGGYDTAAAGNG–3') and 802R (5'–TACNVGGGTATCTAATCC–3'; Lu et al., 2018; Zhu et al., 2018). The DNA libraries were constructed using a TruSeq Nano DNA LT Library Prep Kit (Illumina, San Diego, CA, United States) following the preparation guide, and the amplicons were sequenced on the Illumina Miseq PE300 Platform.

### Sequence Assignment and Data Analysis

All sequence analyses were performed using fast length adjustment of SHort reads version 1.2.7 (FLASH) and quantitative insights into microbial ecology version 1.8.0 (QIIME; Caporaso et al., 2010; Magoc and Salzberg, 2011). A total of 291,315 raw sequences were obtained, and high-quality sequences (quality score≥Q20 and without poly-N strings) longer than 150bp were reserved. The unique operational taxonomic units (OTUs) were clustered at 97% sequence similarity. The OTUs with only one sequence (singleton) were eliminated, and the same number of sequences from each sample were subsampled for further analysis. Classification was carried out using the SILVA database (release 132) with 80% cutoff (Quast et al., 2013). Rarefaction curves based on the identified OTUs were estimated by PAST (version 3.18; Supplementary Figure 2). The Good’s coverage, richness (Chao1), and diversity (Simpson’s and Shannon’s) indexes were calculated using QIIME. Clustering and non-metric multidimensional scaling (NMDS) analysis based on Bray-Curtis similarity of OTUs relative abundance were carried out using PRIMER 6 (version 6.1.16). To determine significant differences in bacterioplankton communities between HC and control and among different sampling days, the analysis of similarity (ANOSIM) was tested using PRIMER 6.

### Statistical Analyses

The significance of differences in Chl *a* content, total bacterioplankton abundance, viral abundance, and Good’s coverage, Chao1, Shannon’s, and Simpson’s indexes between HC and the control were assessed by the paired-samples *t*-test using PASW statistical software (version 18.0.0). To determine the significance of differences in bacterioplankton communities between HC and the control and among different sampling times, statistical analysis of similarities was conducted using PRIMER 6. Significant differences in bacterioplankton community composition between HC and control at the phylum, class, order, family, and genus levels were detected using the statistical analysis of metagenomic profiles (STAMP) software package (Parks et al., 2014). To explore the relationship between bacterioplankton communities and environmental variables (pH, Chl *a*, bacterial abundance, and viral abundance), distance-based multivariate regression analysis (DistLM) was carried out using forward selection in Primer 6 with the PERMANOVA+ add on package (version 1.0.6).

## Results

### Environmental Conditions

The abiotic and biotic characteristics of *in situ* seawater are shown in Supplementary Table 1. The initial pH value of seawater was 7.55, while the mean pH value of global seawater is estimated to be about 8.1. The temperature of the seawater collection was 17.5°C, and this temperature was maintained during the incubation period. The sampling station was located at a subtropical coastal ecosystem that was affected by saline water from the South China Sea. Therefore, the salinity at this location (31.55) was higher than that at other areas around Xiamen (about 23.86-30.00, unpublished data), and this result was consistent with prior studies (Liu et al., 2017; Wang et al., 2019). The collected seawater had an initial dissolved oxygen concentration of 8.68mgl^−1^. Similar to the results of other studies, the seawater was eutrophic and the nutrient concentrations of NO_2_^−^, NO_3_^−^, PO_4_^3−^, and SiO_3_^2−^ were 7.11, 51.66, 1.38, and 34.40μmoll^−1^, respectively (Chen et al., 2019; Wang et al., 2019). The abundance of *in situ* bacterioplankton (1.43±0.07×10^6^ cells ml^−1^) at the sampling station was consistent with published values for Xiamen Bay (Wang et al., 2019). The abundance of *Synechococcus* and picoeukaryotes was 2.40±0.15×10^3^ cells ml^−1^ and 1.09±0.00×10^4^ cells ml^−1^, respectively. In addition, no *Prochlorococcus* was detected at the sampling site.

### Dynamics of Bacterioplankton Abundance

**Figure 2:**
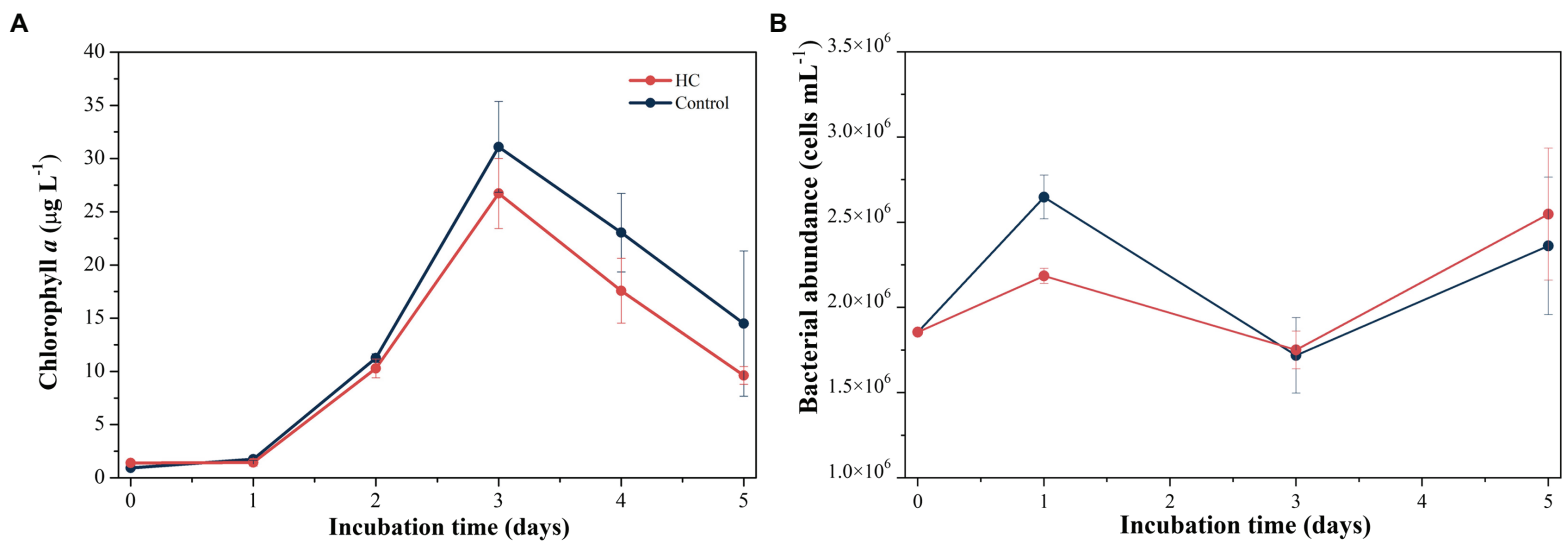
Changes in Chl *a* concentration **(A)** and total bacterioplankton abundance **(B)** during incubation under elevated *p*CO_2_ (HC, about 1,000 μatm) or ambient *p*CO_2_ (Control, about 380–410 μatm) conditions in microcosm experiment. Error bars indicated SD of replicate samples.

During the 5-day incubation period, phytoplankton abundance was assessed by the Chl *a* concentration, which increased from 1.39±0.00μgl^−1^ to 26.70±3.29μgl^−1^ in HC and from 0.93±0.00μgl^−1^ to 31.10±4.27μgl^−1^ in the control in the first 3-days and declined afterward (Figure 2A), and no significant difference in Chl *a* between HC and the control was found (*t*-test, *p*>0.05). The abundance of total bacterioplankton increased on day 1, dropped to the lowest values of 1.75±0.11×10^6^ cells ml^−1^ in HC and 1.72±0.22×10^6^ cells ml^−1^ in the control on day 3, and then slightly increased toward the end of the incubation period (Figure 2B). In addition, bacterial abundance was insensitive to elevated *p*CO_2_, although it was slightly higher in HC than in the control on day 1 of incubation. The bacterioplankton grew preferentially on the first day but not keep increasing concomitant with phytoplankton in the following days, and nutrients competition may be one of the reasons for the different growth patterns of bacterioplankton and phytoplankton (Thingstad et al., 2008; Zhang et al., 2017; Huang et al., 2018). During the incubation period, viral abundance in both HC and the control remained nearly constant with a range of 1.16–1.91×10^7^ particles ml^−1^, and no significant difference between HC and the control was observed (*t*-test, *p*>0.05).

### Bacterioplankton Community Composition

**Figure 3:**
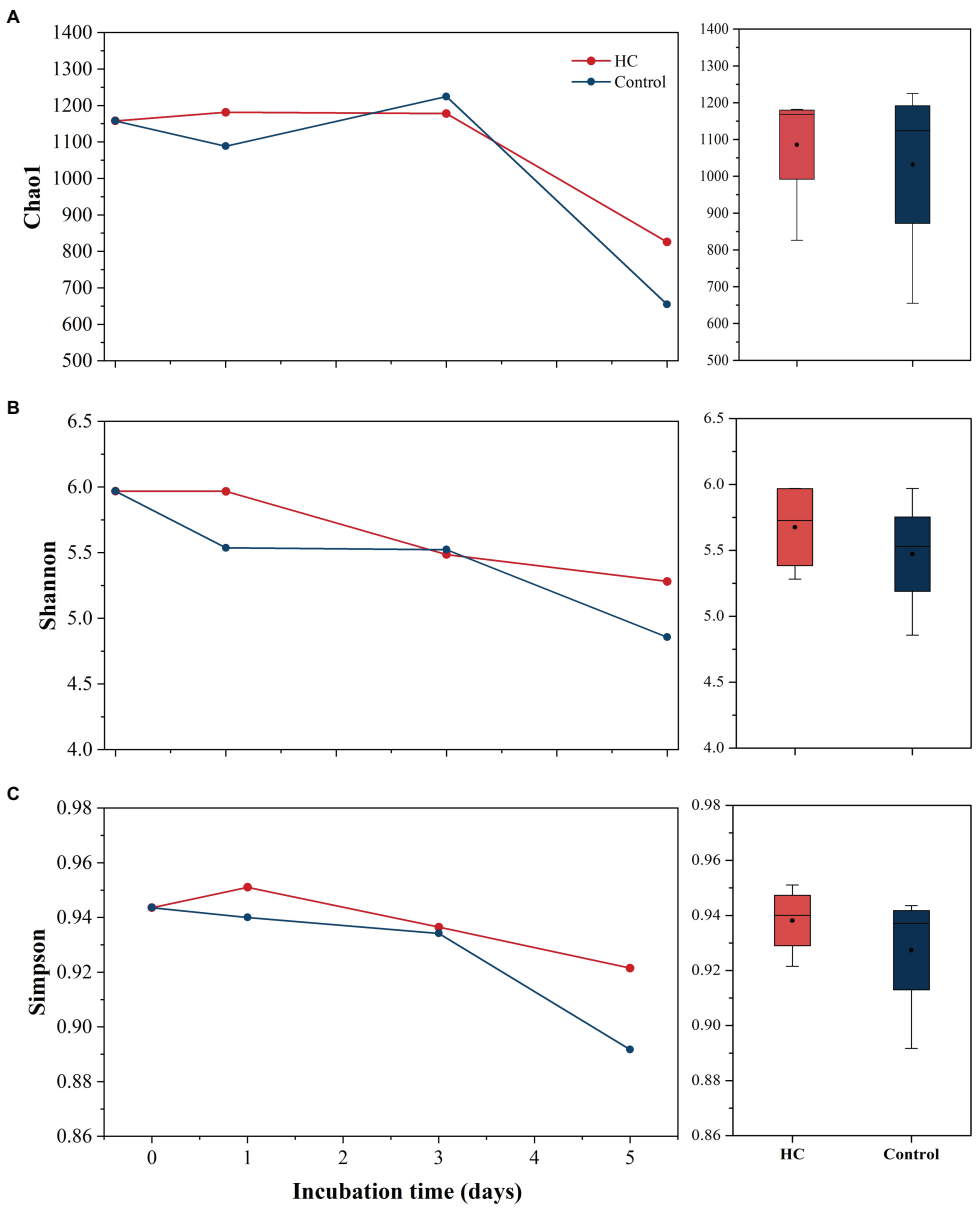
Bacterioplankton community richness **(A)** and diversity **(B,C)** in elevated *p*CO_2_ (HC, about 1,000 μatm) and ambient *p*CO_2_ (Control, about 380–410 μatm) conditions. Changes in Chao1 index, Simpson’s index, and Shannon’s index over time are shown on the left. Boxplots on the right show responses of Chao1 index, Simpson’s index, and Shannon’s index to elevated *p*CO_2_ throughout the whole experiment.

**Figure 4:**
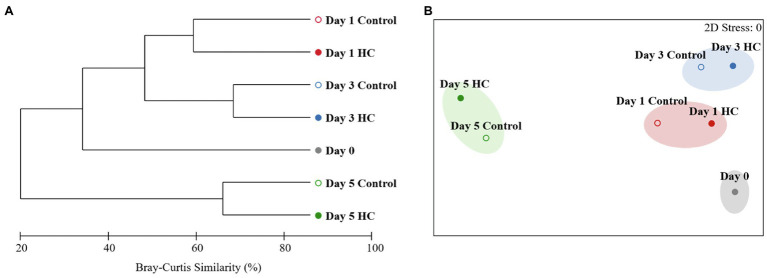
Clustering **(A)** and non-metric multidimensional scaling (NMDS; **B)** analyses showing relationships between the bacterioplankton communities in elevated *p*CO_2_ (HC, about 1,000 μatm, solid circles) and ambient *p*CO_2_ (Control, about 380–410 μatm, hollow circles) conditions during microcosm experiment. Similarities were based on Bray-Curtis similarity. Samples were clustered at a similarity level of 70% in NMDS analysis.

After screening and quality control, a total of 230,562 high-quality sequences were obtained from all samples. Subsamples with 8,832 sequences from each sample were clustered into 403 to 686 OTUs at the 97% sequence similarity level (Supplementary Table 2). The richness of the bacterioplankton community was found to be invariable in the first 3-days but decreased to 71.33% in HC and 56.53% in the control on day 5 of the incubation period. Although the bacterioplankton community richness did change during the incubation period, it did not significantly differ between HC and the control (*t*-test, *p*>0.05; Figure 3). Likewise, bacterioplankton community diversity, as indicated by Shannon’s and Simpson’s indexes, decreased slightly on the last day and was not affected by elevated *p*CO_2_ (*t*-test, *p*>0.05; Figure 3). The results of the clustering and NMDS analyses showed that the bacterioplankton community composition changed during the incubation and was significantly different on day 5 of the incubation period than on other days (Figure 4). Further analyses revealed that the temporal shift in bacterioplankton community composition was significant (ANOSIM global test: global *R*=1, *p*=0.01). However, there was no evidence that elevated *p*CO_2_ affect the bacterioplankton community composition (ANOSIM global test: global *R*=−0.222, *p*>0.05).

**Figure 5:**
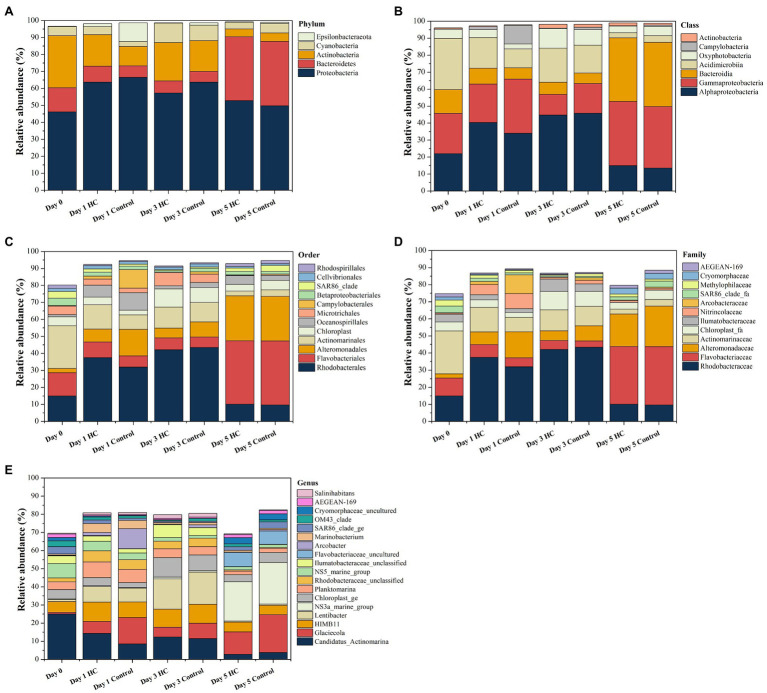
Composition of bacterioplankton community at the phylum **(A)**, class **(B)**, order **(C)**, family **(D),** and genus **(E)** levels in elevated *p*CO_2_ (HC, about 1,000 μatm) and ambient *p*CO_2_ (Control, about 380–410 μatm) conditions during incubation. Charts show relative abundance of taxa present at >1%.

**Figure 6:**
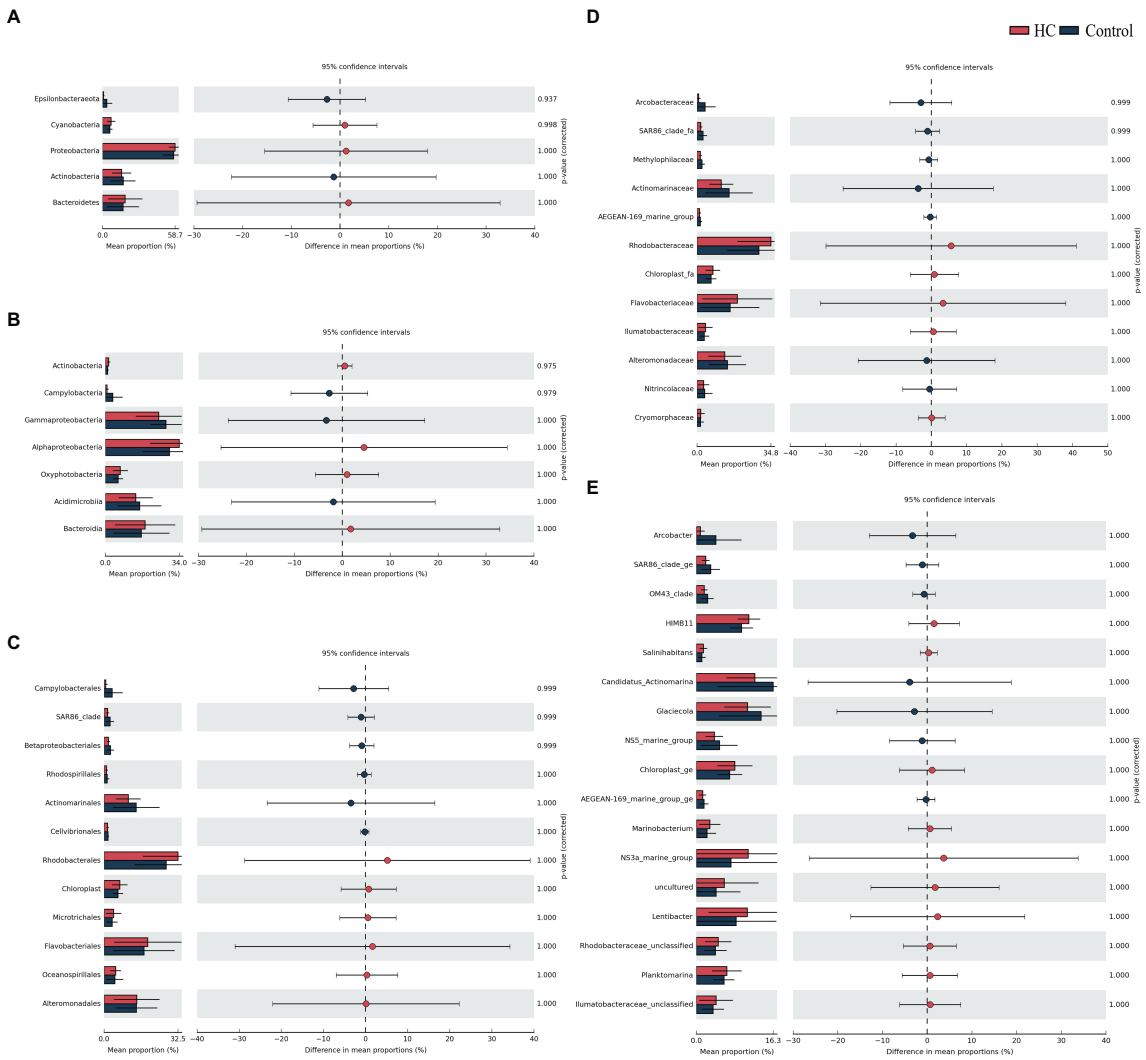
STAMP analysis of relative abundance of bacterioplankton communities at the phylum **(A)**, class **(B)**, order **(C)**, family **(D),** and genus **(E)** levels between elevated *p*CO_2_ (HC, about 1,000 μatm) and ambient *p*CO_2_ (Control) conditions.

Overall, bacterioplankton communities were allocated to 26, 44, 125, 198, and 372 groups at the phylum, class, order, family, and genus levels, respectively (Supplementary Table 3). Bacterioplankton community compositions at the phylum, class, order, family, and genus levels (with relative abundance higher than 1%) are shown in Figure 5. Proteobacteria, which mainly consisted of Alphaproteobacteria and Gammaproteobacteria, were predominant in both HC and the control throughout the incubation period, accounting for about 57.17% of the bacterioplankton community (Figure 5). Bacteroidetes, Actinobacteria, Cyanobacteria, and Epsilonbacteraeota were also abundant in all microcosms and were not affected by elevated *p*CO_2_ (Figures 5, 6). The relative abundance of Bacteroidetes increased by about three-fold during the incubation period, while the proportion of Actinobacteria decreased to 16.67% (Supplementary Figure 3). As expected, Cyanobacteria reached to the highest abundance after 3-day incubation and were insensitive to elevated *p*CO_2_, consistent with the Chl *a* concentration (Supplementary Figure 3; Figure 6). Likewise, bacterial groups at finer levels varied over time but did not differ significantly between HC and the control (STAMP analysis, *p*>0.05; Figures 5, 6). These results suggested that dominant groups constantly changed over time resulting in a prominent temporal shift in the bacterioplankton community. However, contrary to our expectation, the relative abundance of these taxa did not differ significantly between HC and the control (*t*-test, *p*>0.05), indicating that the bacterioplankton community from the Xiamen coastal ecosystem remained stable under elevated *p*CO_2_ conditions upon a short-term incubation.

To identify the potential drivers of changes in the bacterioplankton community, DistLM-forward analysis was carried out to explore the relationships between environmental variables and the bacterioplankton community. However, the bacterioplankton community was not significantly correlated with any of the variables of the microcosms in this study, indicating that these variables could not explain the changes in the bacterioplankton community during the incubation period (Table 1).

**Table 1:** Relationship between bacterioplankton communities and environmental variables (pH, Chl *a*, bacterial abundance and viral abundance) in microcosms as determined by distance-based multivariate regression analysis with forward selection (DistLM-forward).

Variables	Pseudo-F	*P*	*r* ^2^	Prop.	Cumulative
Chl *a*	1.6012	0.1701	0.2429	0.2426	0.2426
Bacterial abundance	2.1094	0.1233	0.5041	0.2615	0.5041
pH	0.8383	0.5331	0.6124	0.1083	0.6124
Viral abundance	0.6514	0.6497	0.7076	0.0952	0.7076

*The response variables were transformed in log (X+1) and then converted into Euclidian distance similarities matrices. The Pseudo-F and the values of p were obtained by permutation (n=9,999)*.

## Discussion

Our results showed that both the population size and community structure of bacterioplankton were not significantly affected by elevated *p*CO_2_ during 5-days of incubation, indicating that the bacterioplankton community in the coastal Xiamen Bay ecosystem was adaptable to the short-term elevated *p*CO_2_. Similar results were also observed in earlier investigations (Grossart et al., 2006; Newbold et al., 2012; Oliver et al., 2014). For instance, elevated *p*CO_2_ did not significantly affect the total bacterial cell count distributions in a marine picoplankton community under phytoplankton pre-bloom and post-bloom conditions (Newbold et al., 2012). Bacterioplankton communities were found to be highly resistant to short-term catastrophic *p*CO_2_ perturbation in a mesocosm experiment, and no significant differences in community abundance, structure, or composition were observed (Oliver et al., 2014).

Recently, a hypothesis was re-proposed that environmental stability might influence the sensitivity of the bacterioplankton community to climate change (Liu et al., 2010; Joint et al., 2011; Wang et al., 2021). In other words, many bacterioplankton communities are already adapted to changing environments due to long-term exposure to variable environmental conditions and subsequent influence. The evidence to date suggests that coastal communities might be more resistant or flexible under changing environmental conditions than communities in more stable environments such as open ocean gyres (Wang et al., 2021). In addition, many studies have detected minor effects of elevated *p*CO_2_ on bacterial abundance, while only few studies showed statistical significant responses which were mainly conducted in oligotrophic oceans, implying that nutrients might be an important influence on the relationship between bacterial population size and elevated *p*CO_2_ (Grossart et al., 2006; Allgaier et al., 2008; Newbold et al., 2012; Maas et al., 2013; Bergen et al., 2016; Lin et al., 2018; James et al., 2019; Xia et al., 2019; Allen et al., 2020; Crummett, 2020; Hu et al., 2021). Similarly, significant impacts of elevated *p*CO_2_ on bacterioplankton community diversity and composition also have been reported to be associated with nutrient regimes (Roy et al., 2013; Sala and Aparicio, 2016; Allen et al., 2020). By way of illustration, bacterioplankton community composition changed consistently in response to elevated *p*CO_2_ at the ultra-oligotrophic center of the South Pacific gyre, while no significant *p*CO_2_ treatment effect was found at the mesotrophic fringe of the South Pacific gyre (Allen et al., 2020). In general, therefore, it seems that bacterioplankton communities from coastal ecosystems were more stable in response to elevated *p*CO_2_ and these ecosystems were always characterized as rapidly changing and eutrophic.

The Xiamen coastal ecosystem is a typical subtropical coastal ecosystem that is subjected to complex geographical and environmental influences. One of the consequences caused by anthropogenic activities and hydrological factors was the drastic pH fluctuation in Xiamen coastal ecosystem. The highest pH value of Xiamen coastal water has exceeded 8.5, and the lowest was under 7.5 for the last few decades (Cai et al., 2016). In addition, the probability of acid rain in Xiamen reached up to 68.8%, and this might also contribute to the low pH of the seawater.^1^ Therefore, the bacterioplankton community in the Xiamen coastal region has already experienced the average surface ocean pH predicted to occur at the end of the century or even lower. Previous studies showed that the bacterioplankton community composition in an elevated *p*CO_2_ mesocosm would be more conserved through time and resistant to CO_2_ perturbation (Oliver et al., 2014). In addition, the variable pH in coastal ecosystems should also consider the effects of biological activity although the changes might be contrary to expectation in the future ocean. Biological driven diel fluctuations in pH could reach to 0.3–0.5 pH units in coastal ecosystems and even exceed 0.5 pH units during phytoplankton blooms or red tides (Joint et al., 2011; Hendriks et al., 2015). According to the records of red tide outbreak in the Xiamen coastal ecosystem, a total of 53 red tide events were recorded from 1986 to 2017 (Chen et al., 2021a). All these evidence indicated that the Xiamen coastal seawater has undergo drastic pH fluctuations on daily, seasonal, and even inter-annual scales. The coastal bacterioplankton communities in this area have probably adapted to these changes through processes including physiological acclimation and evolution (Evans and Hofmann, 2012). Moreover, as a result of the complexity and fluctuation in coastal habitats, bacterioplankton communities in coastal ecosystems were suggested to be highly variable reflecting the heterogeneity. And this heterogeneity might play a role in community stability (Zinger et al., 2011; Shade et al., 2012).

Prior studies have also noted the importance of trophic states in the response of the bacterioplankton community to ocean acidification. Bacterioplankton communities were suggested to be more resistant to ocean acidification in nutrient-rich waters (Roy et al., 2013; Sala and Aparicio, 2016; Allen et al., 2020). There was an experimental demonstration of the trophic effect in response of bacterioplankton to elevated *p*CO_2_ revealed that more pronounced pH homeostasis genes were aroused to cope with pH stress in oligotrophic marine environments compared with high-nutrient conditions (Bunse et al., 2016). Additionally, the high expression levels of pH homeostasis genes in some bacterial groups are at the expense of growth, and this can ultimately affect the composition and diversity of the bacterioplankton community (Bunse et al., 2016; Allen et al., 2020). However, the energy cost of pH homeostasis expression was not necessary for bacterial cells in eutrophic oceans. The evidence thus far supports the idea that physiological acclimation of the bacterioplankton community to elevated *p*CO_2_ is highly possible in eutrophic and highly changeable primitive environments.

Our results differ from those of a previous study on phytoplankton from the Xiamen nearshore, which showed that CO_2_ enrichment enhanced the relative abundance of Flavobacteria during the early stage of a phytoplankton bloom (Lin et al., 2018). Notably, we used *in situ* bacterioplankton communities in our study, while the previous study introduced an artificial phytoplankton community into mesocosms system and conducted the incubation for a longer period (Lin et al., 2018). Therefore, one explanation for the differences in results might be that our 5-day incubation was too short to detect the long-term responses of the bacterioplankton community to seawater acidification, since Flavobacteria only showed increased relative abundance in the HC treatments at day 10 in the study of Lin et al. (2018). Another possible explanation is that artificial phytoplankton inoculated in the mesocosms influenced the competitive ability of Flavobacteria group at high *p*CO_2_ level. In addition, the possible interference of bacterial community (including Flavobacteria) of the inoculated phytoplankton cultures could not be ruled out although the authors thought natural bacterioplankton was the determiner of responses to different CO_2_ concentrations.

## Conclusion

Bacterioplankton communities response to elevated *p*CO_2_ in coastal regions are supposed to be foresight and important. Our results suggest the bacterioplankton community in the coastal region of Xiamen appears to be adaptable to the short-term elevated *p*CO_2_ on account of the eutrophic and changeable habitat. To understand the ecological processes and mechanism underlined these phenomena, better experimental setup (e.g., >3 replicates), more comprehensive analysis of relevant environmental parameters (such as dynamics of organic and inorganic nutrients) and including other ecological components (e.g., heterotrophic nanoflagellates and phytoplankton) are required. In addition, given the influences of long-term environmental exposures on microbial phenotypic plasticity, acclimation, and evolutionary adaptation, this study cannot rule out the long-term effects of ocean acidification on coastal bacterioplankton communities. Thus, further experimentation at multiple temporal scales are needed to address issues related to acclimation and adaptation. Considering the diversification of coastal marine ecosystems caused by specific hydrogeological conditions and anthropogenic activities, predicting how coastal bacterioplankton communities will respond to elevated *p*CO_2_ requires more investigations in more coastal ecosystems. Overall, the findings of this study contribute to our knowledge of bacterioplankton community responses to ocean acidification in coastal area and highlight the need for further research toward to understanding the long-term effects of ocean acidification on dynamic coastal ecosystems.

## Data Availability Statement

The data presented in the study are deposited in the national center for biotechnology information (NCBI) sequence read archive (SRA) repository, accession numbers SRR14766467–SRR14766473 (BioProject accession number PRJNA736025; BioSample accession numbers SAMN19606215–SAMN19606221). The names of the repository/repositories and accession number(s) can be found at: https://www.ncbi.nlm.nih.gov/sra/PRJNA736025, PRJNA736025.

## Author Contributions

RZ and NJ supervised the project and revised manuscript. FZ and YY performed the experiments. YY, FZ, XC, HL, and RZ analyzed data and wrote the manuscript. All authors interpreted the data and gave comments on the manuscript.

## Funding

This study was supported by the National Key Research and Development Program of China (2020YFA0608300, 2021YFE0193000), National Natural Science Foundation (41861144018), China Postdoctoral Science Foundation (2019 M662237), the Senior User Project of RV KEXUE (KEXUE2020G10) from Center for Ocean Mega-Science, Chinese Academy of Sciences, and China Scholarship Council.

## Conflict of Interest

The authors declare that the research was conducted in the absence of any commercial or financial relationships that could be construed as a potential conflict of interest.

## Publisher’s Note

All claims expressed in this article are solely those of the authors and do not necessarily represent those of their affiliated organizations, or those of the publisher, the editors and the reviewers. Any product that may be evaluated in this article, or claim that may be made by its manufacturer, is not guaranteed or endorsed by the publisher.

## Footnotes

^1^ http://sthjj.xm.gov.cn/zwgk/ghcw/hjzlgb/201501/t20150112_1033710.htm
