# Supplementary material for: Insignificant Response of Bacterioplankton Community to Elevated pCO2 During a Short-Term Microcosm Experiment in a Subtropical Eutrophic Coastal Ecosystem
Source: Front Microbiol. 2021 Nov 12;12:730377. doi: 10.3389/fmicb.2021.730377 (PMC8633418; doi:10.3389/fmicb.2021.730377)

SUPPLEMENTARY MATERIALS

Supplementary Figure 1 | Changes in seawater pH during the incubation period.

Supplementary Figure 2 | Rarefaction analysis curve of OTUs based on 16S rRNA sequences at 97% similarity threshold.

Supplementary Figure 3 | Relative abundance of bacterioplankton taxa (relative abundance > 1%) at the phylum levels under elevated pCO_2 (HC) and ambient pCO_2 (control) conditions during incubation.

Supplementary Figure 1

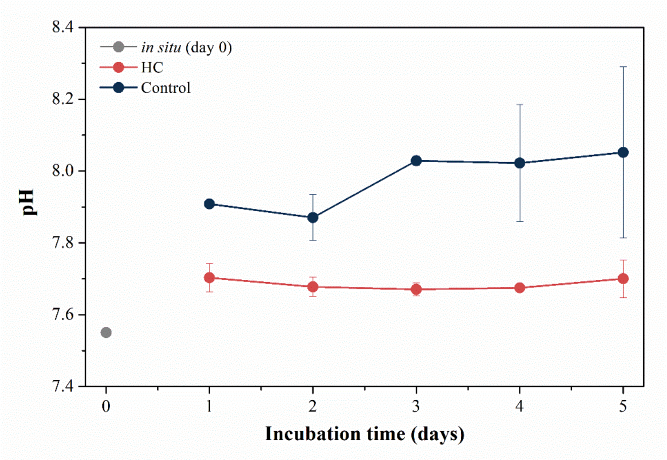

Supplementary Figure 2

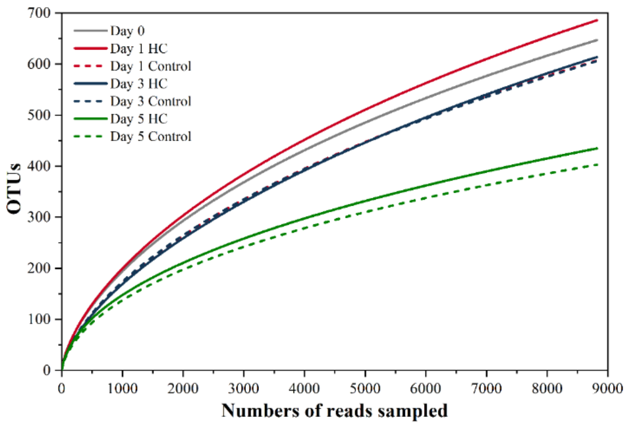

Supplementary Figure 3

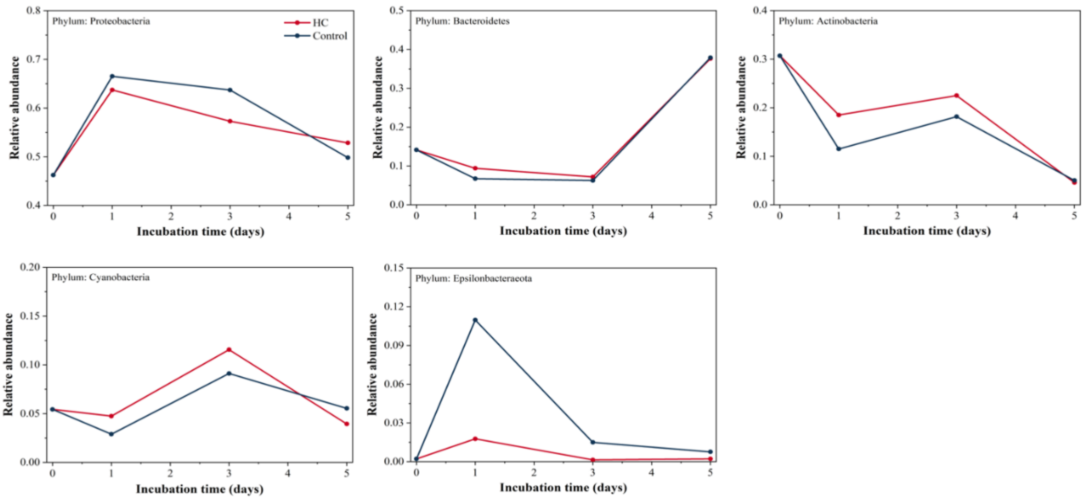

Supplement: Supplementary file 1 [file Data_Sheet_1.zip › Image S1-S3.pdf]
