# Supplementary material for: Insignificant Response of Bacterioplankton Community to Elevated pCO2 During a Short-Term Microcosm Experiment in a Subtropical Eutrophic Coastal Ecosystem
Source: Front Microbiol. 2021 Nov 12;12:730377. doi: 10.3389/fmicb.2021.730377 (PMC8633418; doi:10.3389/fmicb.2021.730377)
Supplement: Supplementary file 1 [file Data_Sheet_1.zip › Table S1-S3.pdf]

SUPPLEMENTARY MATERIALS

Supplementary Table 1 | Abiotic and biotic parameters measured at the sampling station before incubation.

Supplementary Table 2 | Sequence information for samples from the elevated pCO_2 treatments (HC) and ambient pCO_2 treatments (control) during incubation.

Supplementary Table 3 | Numbers of taxa at phylum, class, order, family and genus levels in each sample.

Supplementary Table 1

In-situ Parameters	Data
Longitude	118°14'11.61" E
Latitude	24°29'46.53" N
Depth (m)	1
pH	7.55
Dissolved oxygen (mg L^{-1})	8.68
Temperature (℃)	17.5
Salinity	31.55
NO_2^- (μmol L^{-1})	7.11
NO_3^- (μmol L^{-1})	51.66
NO_2^-+NO_3^- (μmol L^{-1})	58.77
PO_4^{3-} (μmol L^{-1})	1.38
SiO_3^{2-} (μmol L^{-1})	34.4
Synechocuccus (cells mL^{-1})	$2.40±0.15×10^3$
Picoeukaryote (cells mL^{-1})	$1.09±0.00×10^4$
Bacteria (cells mL^{-1})	$1.43±0.07×10^6$

Supplementary Table 2

Samples	Raw Sequences	High-quality Sequences	High-quality Sequences Percent (%)	Subsampled Sequences	No. of OTUs
Day 0	43758	28304	0.6468	8832	647
Day 1 HC	28204	18404	0.6525	8832	686
Day 1 Control	30447	19955	0.6554	8832	607
Day 3 HC	57228	45116	0.7884	8832	614
Day 3 Control	31759	26269	0.8271	8832	606
Day 5 HC	19121	17611	0.921	8832	435
Day 5 Control	80798	74903	0.927	8832	403

Supplementary Table 3

Samples	Phylum	Class	Order	Family	Genus
Day 0	20	29	94	129	191
Day 1 HC	15	23	75	110	186
Day 1 Control	15	23	69	99	172
Day 3 HC	16	27	79	117	199
Day 3 Control	15	23	76	114	195
Day 5 HC	18	27	75	114	209
Day 5 Control	14	21	63	103	182
Total	26	44	125	198	372
